# Immune Checkpoint Inhibition for Triple-Negative Breast Cancer: Current Landscape and Future Perspectives

**DOI:** 10.3389/fonc.2021.648139

**Published:** 2021-05-19

**Authors:** Huimei Yi, Ying Li, Yuan Tan, Shujun Fu, Faqing Tang, Xiyun Deng

**Affiliations:** ^1^Key Laboratory of Translational Cancer Stem Cell Research, Hunan Normal University, Changsha, China; ^2^Departments of Pathology and Pathophysiology, Hunan Normal University School of Medicine, Changsha, China; ^3^Hunan Key Laboratory of Oncotarget Gene, Changsha, China; ^4^Department of Clinical Laboratory, Hunan Cancer Hospital & the Affiliated Cancer Hospital of Xiangya School of Medicine, Central South University, Changsha, China

**Keywords:** triple-negative breast cancer, immune checkpoint, PD1/PDL1, CTLA4, Immunotherapy

## Abstract

Triple-negative breast cancer (TNBC) is characterized by the lack of clinically significant levels of estrogen receptor (ER), progesterone receptor (PR), and human epidermal growth factor receptor 2 (HER2). Owing to the aggressive nature and the emergence of resistance to chemotherapeutic drugs, patients with TNBC have a worse prognosis than other subtypes of breast cancer. Currently, immunotherapy using checkpoint blockade has been shown to produce unprecedented rates of long-lasting responses in patients with a variety of cancers. Although breast tumors, in general, are not highly immunogenic, TNBC has a higher level of lymphocyte infiltration, suggesting that TNBC patients may be more responsive to immunotherapy. The identification/characterization of immune checkpoint molecules, i.e., programmed cell death protein 1 (PD1), programmed cell death ligand 1 (PDL1), and cytotoxic T lymphocyte-associated antigen 4 (CTLA4), represents a major advancement in the field of cancer immunotherapy. These molecules function to suppress signals downstream of T cell receptor (TCR) activation, leading to elimination of cytotoxic T lymphocytes (CTLs) and suppression of anti-tumor immunity. For TNBC, which has not seen substantial advances in clinical management for decades, immune checkpoint inhibition offers the opportunity of durable response and potential long-term benefit. In clinical investigations, immune checkpoint inhibition has yielded promising results in patients with early-stage as well as advanced TNBC. This review summarizes the recent development of immune checkpoint inhibition in TNBC, focusing on humanized antibodies targeting the PD1/PDL1 and the CTLA4 pathways.

## Introduction

Triple-negative breast cancer (TNBC), accounting for about 10–20% of all breast cancer cases, is the most aggressive and fatal subtype of breast cancer (1, 2). Compared with other subtypes, TNBC cases are more prevalent in women of African ancestry and tend to be younger at diagnosis (3). Due to the lack of clinically significant levels of estrogen receptor (ER), progesterone receptor (PR), and human epidermal growth factor receptor 2 (HER2), there is no effective targeted therapeutic agent currently available for TNBC. At present, chemotherapy remains the mainstay of systemic treatment in TNBC (4). Resulting from the emergence of resistance to chemotherapeutic drugs, TNBC patients have a worse prognosis than patients with receptor-positive breast cancer, with a median overall survival (OS) of ≤ 18 months (5, 6). Nowadays, inhibitors of poly (ADP-ribose) polymerases (PARPs) have been approved for a proportion of TNBC patients, i.e., those with BRCA mutation (7). Obviously, more effective treatment modalities are needed to improve the prognosis of this subtype of breast cancer.

Unlike other cancer types that respond well to immunotherapy, most breast cancers are not inherently immunogenic and typically have a low level of lymphocyte infiltration. However, as a special subtype with poorer prognosis, TNBC has greater tumor immune infiltrate, which is characterized by a higher number of tumor-infiltrating lymphocytes (TILs). Clinical investigations have shown that a higher percentage of CD8^+^ cytotoxic T lymphocytes (CTLs) is a feature associated with higher response rates to immune checkpoint inhibition and can predict favorable survival outcomes in TNBC patients (8, 9). Based upon the findings of a phase III clinical trial IMpassion130 (10), the US FDA granted accelerated approval to the immune-chemotherapy combination of an anti-programmed cell death ligand 1 (anti-PDL1) antibody (atezolizumab) and chemotherapy for PDL1-positive metastatic TNBC (11).

## Immune Checkpoints as Important Targets of Anti-Cancer Therapy

**Figure 1:**
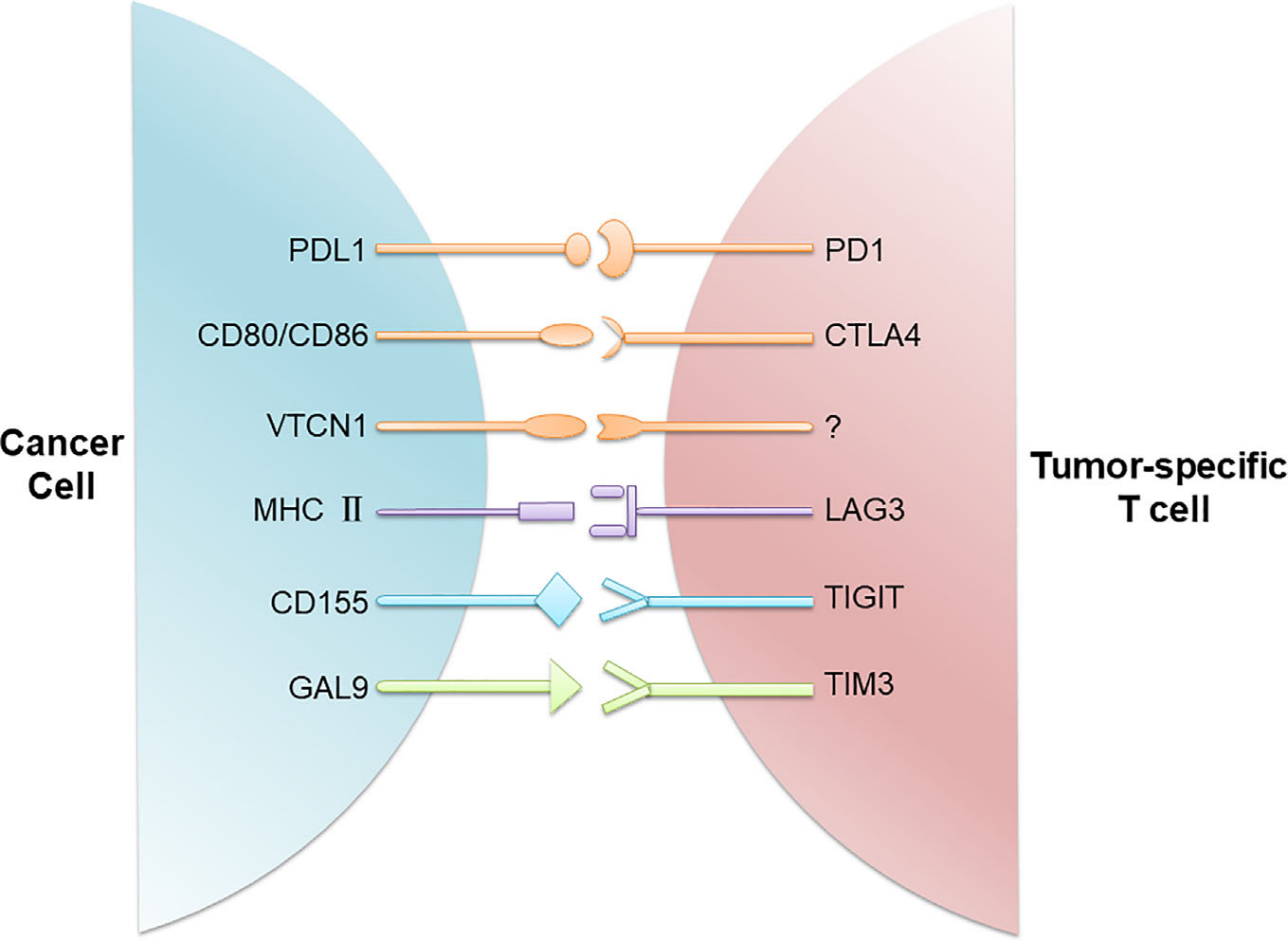
Immune checkpoints involved in T cell inactivation. Cancer cells evade the host immune system through upregulation of immune checkpoints composed of the ligands on the cancer cell and the respective receptors on the CD8^+^ T cell. These ligand/receptor pairs include PDL1/PD1, CD80/CD86/CTLA4, MHC II/LAG3, CD155/TIGIT, and GAL9/TIM3. In addition, VTCN1 is also found on TNBC cells, although its receptor on T cells is not known.

**Figure 2:**
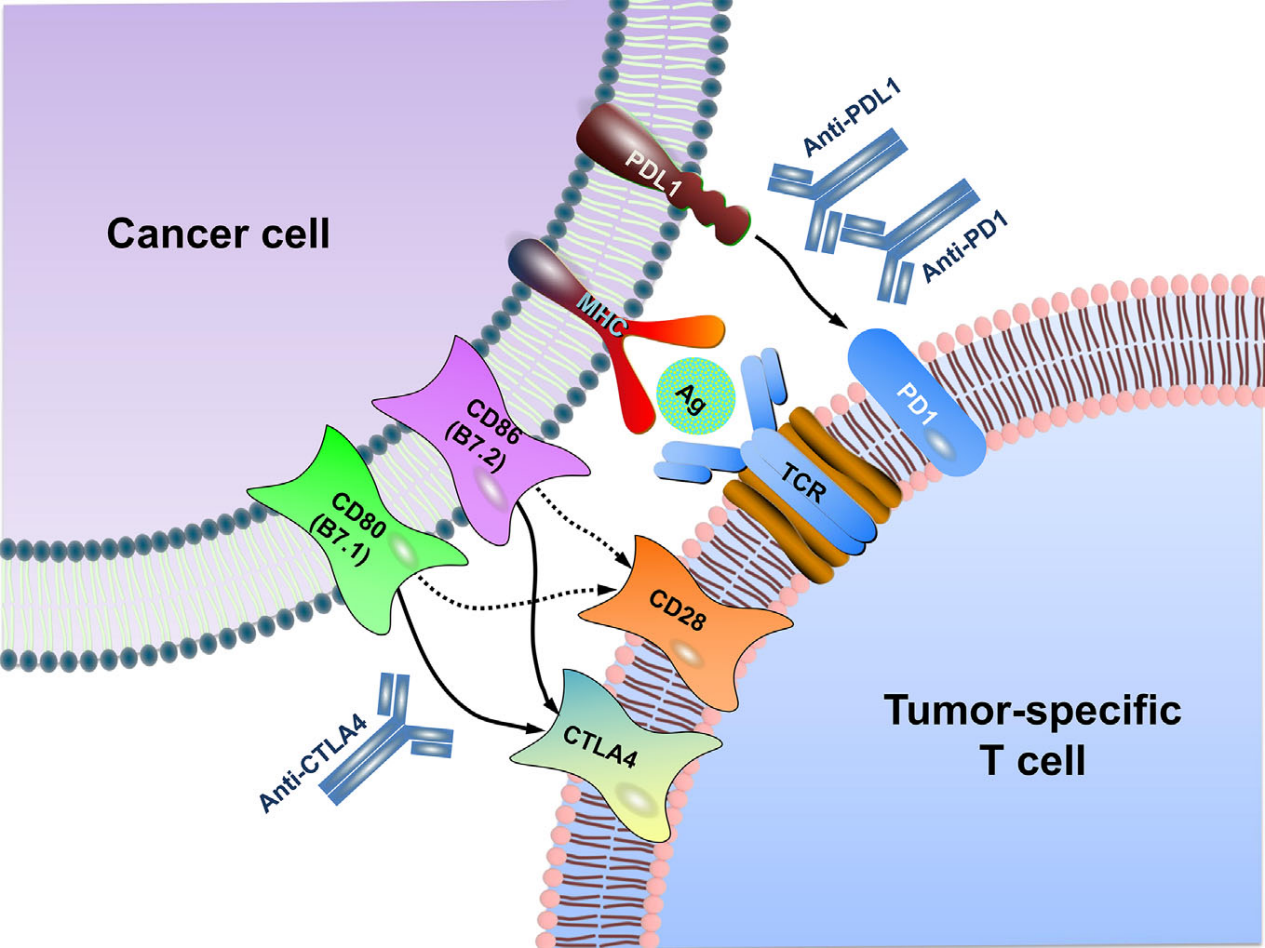
Schematic diagram of immune checkpoint blockade. MHC generally presents antigen on the surface of cancer cells for recognition by CD8^+^ T cells *via* their TCR. CTLA4, as a negative regulator, is homologous to the T cell co-stimulatory protein CD28, both of which bind to CD80 and CD86 on the surface of cancer cell but with different affinity. Overall, CTLA4 has a much higher affinity than CD28 to CD80/CD86. PD1 is expressed on T lymphocyte surface. The binding of PD1 on the T cell with PDL1 functions to suppress signals downstream of TCR activation, leading to apoptosis of the CTL. Antibodies (anti-CTLA4, anti-PD1, anti-PDL1) inhibit these checkpoint targeting proteins to restore the activity of T cells and kill cancer cells. MHC, major histocompatibility complex; TCR, T cell receptor; Ag, antigen.

Immune checkpoints refer to a plethora of inhibitory mechanisms hardwired into the surfaces of tumor cells and immune cells that are crucial for modulating the level and duration of anti-tumor immune responses. These checkpoints are composed of the ligands on the cancer cell and the respective receptors on the CD8^+^ T cell. The ligands expressed on the cancer cell include PDL1, CD80/CD86, major histocompatibility complex class II (MHC II), CD155, and galectin-9 (GAL9), while their corresponding receptors on the CD8^+^ T cell include programmed cell death protein 1 (PD1), cytotoxic T lymphocyte-associated antigen 4 (CTLA4), lymphocyte activating gene 3 (LAG3), T-cell immunoreceptor with immunoglobulin (Ig) and ITIM domains (TIGIT), T cell immunoglobulin and mucin-3 (TIM3), etc. In addition, there is evidence that V-set domain containing T-cell activation inhibitor 1 (VTCN1) also has an important tumor immunosuppressive effect, but its corresponding ligand is not clear yet (12) (**Figure 1**). Activation of the immune checkpoints involves interactions of the inhibitory ligand-receptor molecules. The three most important checkpoint molecules currently used for drug development include PD1, PDL1, and CTLA4 (**Figure 2**).

Up to now, a total of seven antibodies including two anti-PD1 antibodies, three anti-PDL1 antibodies, and two anti-CTLA4 antibodies, have been approved by the FDA for medical use (**Table 1**). In recognition of the eminent contribution to the field of immune checkpoints, the 2018 Nobel Prize in Physiology or Medicine was awarded to James P. Allison at the University of Texas MD Anderson Cancer Center and Tasuku Honjo at Kyoto University. Their seminal work led to the development of antibody-based immune checkpoint inhibitors and the designing of the strategies for activating the anti-tumor immunity in cancer therapy (13).

**Table 1:** Summary of immune checkpoint-targeting antibodies.

Target	Antibody	Trade name	Isotype	Initial approval time
PD1	Pembrolizumab	Keytruda	IgG4	Sep 05, 2014
	Nivolumab	Opdivo	IgG4	Jun 22, 2015
PDL1	Atezolizumab	Tecentriq	IgG1	May 18, 2016
	Avelumab	Bavencio	IgG1	Mar 23, 2017
	Durvalumab	Imfinzi	IgG1	May 1, 2017
CTLA4	Ipilimumab	Yervoy	IgG1	Mar 25, 2011
	Tremelimumab	\	IgG2	Apr 15, 2015

## Targeting the PD1/PDL1 Pathway in TNBC

PD1 (also known as CD279), an inhibitory receptor expressed on the surface of CTLs, is emerging as a promising target of immune checkpoint inhibition (14). The primary role of PD1 is to limit T cell activity in peripheral tissues at the time of an inflammatory response to infection, thus limiting autoimmunity (15). The binding of PD1 on T cells with its ligand PDL1 (also known as B7-H1 or CD274) suppresses the signals downstream of T cell receptor (TCR) activation (16, 17). Expression of PDL1 has been found in 40–60% of all breast tumors and is associated with higher histologic grades, larger tumor sizes, and triple-negative status, all of which are independent indicators of poor prognosis in breast cancer (18–20).

Immune checkpoint inhibition using the antibodies against the PDL1/PD1 pathway has shed light on TNBC. The stages of development of anti-PD1 or anti-PDL1 antibodies and their respective combinatorial agents used in clinical trials of TNBC are summarized in **Table 2**. Particularly, the clinical benefit of TNBC has been derived from the combination of immunotherapy with radiotherapy or chemotherapy (21). Theoretically and practically, these combinations should increase the mutational load of tumors and optimize the microenvironment, thus priming the tumor for immunotherapy and improving progression-free survival (PFS) of the patients. Indeed, these combinations have significantly enhanced the curative effect on TNBC patients, which will be discussed in more detail below.

**Table 2:** PD1/PDL1 inhibitors in TNBC immunotherapy for clinical trials.

Antibody	Combinatorial agent	Clinical trial ID	Phase	Status
Pembrolizumab	\	NCT02981303	II	Completed
	\	NCT03197389	I	Completed
	\	NCT02447003	II	Completed
	Capecitabine; Eribulin; Gemcitabine; Vinorelbine	NCT02555657	III	Completed
	Nab-paclitaxel; Paclitaxel; Gemcitabine; Carboplatin	NCT02819518	III	Active, not recruiting
	Nab-paclitaxel; Doxorubicin; Cyclophosphamide; Carboplatin; Paclitaxel	NCT02622074	I	Completed
	Carboplatin; Doxorubicin; Cyclophosphamide; Epirubicin; Paclitaxel	NCT03036488	III	Active, not recruiting
	LTX-315	NCT01986426	I	Completed
	Lenvatinib	NCT03797326	II	Recruiting
Nivolumab	TAK-659	NCT02834247	I	Completed
	Doxorubicin; Cyclophosphamide; Cisplatin	NCT02499367	II	Active, not recruiting
Atezolizumab	\	NCT03281954	III	Recruiting
	Nab-Paclitaxel	NCT02425891	III	Active, not recruiting
	Pegylated liposomal doxorubicin; Cyclophosphamide	NCT03164993	II	Recruiting
	Paclitaxel; Doxorubicin or Epirubicin; Cyclophosphamide	NCT03498716	III	Recruiting
	Nab-paclitaxel; Doxorubicin; Cyclophosphamide; Filgrastim; Pegfilgrastim	NCT03197935	III	Active, not recruiting
	Nab-Paclitaxel	NCT01633970	I	Completed
	Gemcitabine; Capecitabine; Carboplatin	NCT03371017	III	Recruiting
Avelumab	\	NCT01772004	I	Completed
Durvalumab	\	NCT02489448	I/II	Active, not recruiting
	Nab-Paclitaxel; Epirubicin; Cyclophosphamide	NCT02685059	II	Completed
	Olaparib	NCT03801369	II	Recruiting
	Cediranib; Olaparib	NCT02484404	I/II	Recruiting
	Cyclophosphamide; Doxorubicin hydrochloride; Paclitaxel	NCT00856492	II	Completed
	Hiltonol	NCT02826434	I	Active, not recruiting

### Anti-PD1 Antibodies

#### Pembrolizumab

As a humanized anti-PD1 antibody that received initial FDA approval for unresectable or metastatic melanoma in 2014, pembrolizumab is one of the best studied immune checkpoint inhibitors (22). In 2016, a phase Ib study (the KEYNOTE-012 trial) reported the efficacy with an acceptable safety profile when pembrolizumab was given to patients with heavily pretreated, advanced TNBC. Among the 27 patients evaluable for anti-tumor activity, the overall response rate was 18.5%, with a median response time of 17.9 weeks (23).

The combined immunotherapy of pembrolizumab and chemotherapy has been investigated in breast cancer. In the locally advanced breast cancer, the addition of pembrolizumab to standard neoadjuvant chemotherapy (paclitaxel followed by doxorubicin and cyclophosphamide) increased the rate of pathological complete response (pCR) by approximately three-fold (60% *vs.* 20%) (24). It is reported that pembrolizumab/chemo combination improves PFS in metastatic TNBC. Results showed that in the intention-to-treat analysis of the full cohort, regardless of PDL1 status, the median PFS was 7.5 months with pembrolizumab and 5.6 months with placebo. The 6-month PFS rates were 55.4% and 47.8%, respectively, and the 12-month PFS rates were 29.8% and 20.9%, respectively (25). Clinical trials of pembrolizumab alone or in combination with different chemotherapeutic agents, monoclonal antibodies, or small molecule inhibitors are now under active investigation in numerous clinical trials in TNBC (**Table 2**).

A strategy of combination of pembrolizumab with PARP inhibitor yielded an objective response rate of 45% compared to 16.7% in single-agent PARP inhibitor group (26). A clinical trial (NCT02555657) aimed to treat metastatic TNBC with pembrolizumab, in which 622 patients were randomly assigned to receive either pembrolizumab or chemotherapy. Median follow-up time was 31.4 months for the pembrolizumab group and 31.5 months for the chemotherapy group. Median OS in patients with PDL1 with combined positive score (CPS) of 10 or more was 12.7 months for the pembrolizumab group and 11.6 months for the chemotherapy group. In the overall population, median OS was 9.9 months for the pembrolizumab group and 10.8 months for the chemotherapy group (27). Another clinical trial funded by Merck Sharp & Dohme (NCT03036488) showed that among patients with early TNBC, the percentage of patients with a pCR was significantly higher among those who received pembrolizumab plus neoadjuvant chemotherapy than those who received placebo plus neoadjuvant chemotherapy (28).

#### Nivolumab

Nivolumab is another humanized anti-PD1 monoclonal antibody. Due to its significant clinical efficacy against several types of malignancies, nivolumab has become one of the most eye-catching checkpoint inhibitors. A clinical trial (NCT02834247) investigated TAK-659, a selective inhibitor of the Syk tyrosine kinase, in combination with nivolumab in patients with metastatic TNBC. The maximum tolerated dose and the overall response rate were determined after the patients received TAK-659 at 60 mg/day in combination with nivolumab at 3 mg/kg. This study has been finished on November 30, 2018, and the specific grouping experiment results are available on ClinicalTrials.gov (13). Some scholars pointed out that previous research has shown that anti-PD (L)1 therapy can induce durable responses in patients with metastatic TNBC, but that the response rate is relatively low, about 5-10%. The TONIC study is a currently ongoing phase II trial for patients with metastatic TNBC. The objective response rate (ORR) per RECIST v1.1 with nivolumab for the whole cohort was 22% and 24% for iRECIST, which included 1 (2%) complete response (CR), and 11 (22%) partial responses (PR). Additionally, stable disease (SD) lasting more than 24 weeks was achieved in 1 (2%) patient, which resulted in a 26% clinical benefit rate. This is the first trial that has shown promising results using nivolumab after giving either radiation or chemotherapy. The completion of this study is estimated to be in August of 2022 (29). In a study published in 2019 (NCT02499367), 67 patients with metastatic TNBC were treated with the anti-PD1 antibody nivolumab after 2 weeks of either hypofractionated irradiation of a single tumor site, low-dose cyclophosphamide, cisplatin, or doxorubicin, or no induction therapy. Overall, the ORR was 20% and, although the median PFS was only 1.9 months, the median duration of response was 9 months (30). Trials of nivolumab alone or in combination with ipilimumab (anti-CTLA4 antibody), different chemotherapeutic agents, monoclonal antibodies, or vascular endothelial growth factor receptor (VEGFR) inhibitor on TNBC are ongoing (**Table 2**).

### Anti-PDL1 Antibodies

#### Atezolizumab

Atezolizumab (MPDL3280A), a humanized monoclonal antibody against PDL1, was reported to significantly increase median OS and objective remission rate in lung cancer patients in a phase II trial (31). In May of 2016, the FDA granted accelerated approval to atezolizumab for the treatment of locally advanced and metastatic tumors (11). An initial phase I study demonstrated that of the nine patients with advanced TNBC evaluated for efficacy of atezolizumab, the overall response rate was 33% (32). Recently, a phase III clinical trial (NCT02425891) evaluating the effects of atezolizumab in combination with nab-paclitaxel as first-line treatment in metastatic TNBC patients yielded exciting results. Among the patients with PDL1-positive tumors, atezolizumab plus nab-paclitaxel significantly prolonged the median OS compared with placebo plus nab-paclitaxel (25.0 *vs.* 15.5 months) (10). It should be noted that as the benefit was observed in the patients with PDL1-expressing tumors (accounting for about 40-60% of all TNBC) (10, 23), the overall effect on TNBC patients as a whole is not satisfactory and still needs improvement. A phase Ib clinical trial (NCT01633970) examined the safety, tolerability, and clinical activity of atezolizumab (one or more doses) plus nab-paclitaxel in 33 patients with metastatic TNBC. All patients experienced at least 1 treatment-related adverse event (AE), 73% patients experienced grade 3/4 AEs, and 21% patients had grade 3/4 AEs of special interest. No death was noted in this study. The ORR was 39.4%, and median PFS and OS were 5.5 months and 14.7 months, respectively (33). As mentioned earlier, the FDA has granted accelerated approval to the combination of atezolizumab with nab-paclitaxel for the treatment of PDL1-positve metastatic TNBC. Ongoing trials in TNBC are using atezolizumab alone or in combination with different chemotherapeutic agents, monoclonal antibodies, or small molecule inhibitors (**Table 2**). These efforts are expected to lead to new treatment options for patients with TNBC in the near future.

#### Avelumab

Avelumab, another anti-PDL1 antibody, was investigated as adjuvant treatment for TNBC in a phase Ib randomized trial (NCT01772004). In this trial, 168 patients with metastatic breast cancer, including 58 patients with TNBC, were included. Patients refractory to or progressing after standard-of-care therapy received avelumab. 13.7% patients had higher than grade 3 AEs, including two deaths. The ORR was 3.0% in all subtypes of breast cancer and 5.2% in TNBC patients. A trend toward a higher ORR was seen in patients with PDL1-positive vs. PDL1-negative tumor-associated immune cells in the overall population (16.7% vs. 1.6%) and in the TNBC subgroup (22.2% vs. 2.6%) (34). Furthermore, avelumab alone or in combination with different chemotherapeutic agents, monoclonal antibodies, or lansoprazole, a proton-pump inhibitor, is currently under investigation in TNBC (**Table 2**).

#### Durvalumab

Several trials are also being performed with durvalumab for patients with metastatic TNBC in combination therapy (19) (**Table 2**). In the GeparNuevo trial, the positive rates of pCR in patients receiving durvalumab treatment 2 weeks before chemotherapy was significantly higher than that in the placebo group (61% *vs.* 41.4%). Less improved response rate of 48.4% was seen in patients receiving durvalumab in conjunction with neoadjuvant GeparNuevo (NCT02685059).

A phase Ib trial (NCT02826434) studied the immunotherapeutic effects with a peptide vaccine, PVX-410, and durvalumab as adjuvant setting in treating stage II or III TNBC. The dose-limiting toxicity of PVX-410 vaccine with durvalumab and the immune response of CD8^+^ CTLs to vaccine-specific peptides were detected after patients received 6 injections of the PVX-410 vaccine with poly-ICLC (a dsRNA analog used as an agonist of Toll-like receptor 3 (TLR3)) every 2 weeks and 2 infusions of durvalumab with the 4^th^ and 6^th^ cycle. Currently, this study is still in progress and should be completed in August of 2022.

In a phase I/II trial (NCT02489448), stage I–III TNBC patients were evaluated in terms of whether they produced a higher pCR with adding durvalumab to nab-paclitaxel weekly and then with dose-dense doxorubicin and cyclophosphamide for 4 cycles compared with chemotherapy alone. Additionally, this trial will also demonstrate whether durvalumab is safe and can be given in the full dose when added to this chemoregimen, and the secondary object is to assess the safety and toxicity of adding durvalumab to nab-paclitaxel followed by adding it to dose-dense doxorubicin/cyclophosphamide. Results showed patients treated at the recommended phase II dose of 10 mg/kg of durvalumab achieved a pCR rate of 44%. Among PDL1 positive patients, the pCR rate was 59% and among PDL1 negative patients, the pCR rate was 32%. No significant difference was observed (*P* = 0.26) (35).

Another randomized phase II study (NCT02685059) was performed to evaluate the efficacy of addition of durvalumab to an anthracycline + taxane-based neoadjuvant therapy in early TNBC. A total of 174 patients were randomized, 117 of whom participated in the window-phase. The pCR rate was 53.4% in the durvalumab group and 44.2% in the placebo group. There was a trend for increased pCR rates in PDL1-positive tumors, which was significant for PDL1-tumor-cell in durvalumab group and for PDL1-immune cell in placebo group (36). Targeted mRNA sequencing was performed in samples from patients with early TNBC of the GeparNuevo trial. Signatures were evaluated to predict response to neoadjuvant PDL1 inhibition in combination with chemotherapy. Two mRNA signatures (G6-Sig and IFN-Sig) were predictive for treatment response in a multivariate model, while a simple metric of two key cytolytic effector transcripts (GZMA and PRF1) predicted pCR in the durvalumab arm, and the proliferation-associated gene signature in the placebo arm. Seven genes were identified highly expressed in responders in the durvalumab arm, but not in the placebo arm. These genes were associated with cellular antigen processing and presentation and IFN signaling (37).

## Targeting the CTLA4 Molecule in TNBC

CTLA4 (also known as CD152), the first co-inhibitory molecule identified and the first immune checkpoint receptor clinically targeted (38), is expressed exclusively on T cells where it primarily regulates the amplitude of early-stage T cell activation. The ligands of CTLA4, i.e., CD80 (also known as B7.1) and CD86 (also known as B7.2), are shared by the co-stimulatory receptor CD28 (39). Compared with CD28, CTLA4 has a much higher overall affinity for both CD80 and CD86 (40). Therefore, the expression of CTLA4 on T cell surface dampens the activation of T cells by outcompeting CD28’s positive co-stimulatory signal. This dominance of negative signals from CTLA4-CD80/CD86 interaction results in reducing T cell proliferation and decreasing IL-2 production (41). The central role of CTLA4 in inhibiting T cell activity is demonstrated by the systemic immune lethal hyperactivation phenotype of CTLA4-knockout mice (42).

### Preclinical Studies of CTLA4 Blockade

As an important strategy of cancer immunotherapy, CTLA4 blockade results in broad enhancement of immune responses that are dependent on helper T cells (43). The strategy of blocking CTLA4 was questioned because of lack of tumor specificity to the expression of CTLA4 ligands and because of the dramatic lethal autoimmune and hyperimmune phenotype of CTLA4-knockout mice. Initially, a high degree of immune toxicity associated with blockade of this receptor was predicted. However, Allison and colleagues used preclinical models to demonstrate that a therapeutic window was indeed achieved when CTLA4 was partially blocked with antibodies against CTLA4 (44). Subsequent studies demonstrated significant anti-tumor responses without overt immune toxicities, when the mice bearing partially immunogenic tumors were treated with CTLA4 antibodies. Poorly immunogenic tumors did not respond to anti-CTLA4 as a single agent but did respond when anti-CTLA4 antibody was combined with a granulocyte-macrophage colony-stimulating factor (GM-CSF)-transduced cellular vaccine (45). These preclinical investigations indicate that antibody-mediated CTLA4 blockage has the potential of clinical application in treating immune-related tumors.

### Humanized Antibodies Against CTLA4

The above preclinical findings encouraged the development and testing of two fully humanized CTLA4 antibodies. Ipilimumab (trade name Yervoy), a monoclonal antibody able to effectively block CTLA4 binding to its ligand, is the first immune checkpoint inhibitor approved by the FDA for clinical use (46). Tremelimumab is another anti-CTLA4 monoclonal antibody. As with virtually all anti-cancer agents, initial testing was as a single agent in patients with advanced melanoma and ovarian cancer that were not responding to conventional therapy (47). Both antibodies produced objective clinical responses in ~10% of patients with melanoma, but immune-related toxicities involving various tissue sites were observed in 25–30% of patients, with colitis being a particularly common concern. The first randomized phase III clinical trial to be completed was for tremelimumab in patients with advanced melanoma. In this trial, 15 mg/kg tremelimumab was given every three months as a single agent and compared with dacarbazine, a standard melanoma chemotherapy treatment. The trial showed no survival benefit with this dose and schedule relative to dacarbazine (48). Currently, anti-CTLA4 immunotherapy is being tested in non-small cell lung cancer and melanoma, with a focus on brain metastases, either as monotherapy or in combination with other therapeutic agents (49, 50). The clinical trials of anti-CTLA4 antibodies in TNBC are still in progress, with no definite results published yet.

## Other Immune Checkpoint Targets in TNBC

While immune checkpoint inhibition through the PD1/PDL1 axis and CTLA4 may still not be satisfactory in TNBC, other molecules such as TIM3, LAG3, and TIGIT are investigated in some studies (51, 52). LAG3, an immunological molecular marker expressed in activated T cells, NK cells, B cells, and plasma cell-like dendritic cells (DCs), is the only known ligand for major histocompatibility complex (MHC) molecules (53, 54). Strikingly, in a mouse model of TNBC, the dual blockade of LAG3 and PD1 was shown to achieve a better anti-tumor effect than either one alone (55). TIM3, a member of the TIM family, and expressed in regulatory T cells, DCs, other lymphocyte subsets, subpopulations of macrophages and monocytes (56). Moreover, tissue microarray showed that high TIM3 expression in TILs was significantly associated with better DFS and OS in TNBC patients (57). Surprisingly, it was found that co-blocking of PD1 and PDL1 can upregulate the co-expression of TIM3 and LAG3 on CD4^+^ CD25^+^ T cells, suggesting that resistance to PD1/PDL1 inhibition may develop through upregulation of other immune checkpoint molecules (58). Further investigation on these immune checkpoint molecules may provide alternative immunotherapeutic strategy for TNBC.

## Treatment-Related Adverse Events for Immune Checkpoint Inhibitors

Safety issue has always been an enormous concern for novel cancer therapeutics. Although the results of immune checkpoint blockade are promising so far in clinical trials, most patients do not show long-lasting remission and some cancers have even become completely refractory. Benefit of immune checkpoint blockade may be achieved at the cost of toxicities, in the form of immune-related AEs, which have been subject of discussion in recent publications (59, 60). In general, immune checkpoint inhibition can be continued in patients with most grade I toxicities. Treatment should be suspended, delayed, or discontinued for higher grade toxicities. In a systematic review and meta-analysis of data from 36 comparative phase II and III trials (n = 15,370), investigators compared the safety profiles of commonly used immune checkpoint inhibitors. Atezolizumab (anti-PDL1 antibody) had the best overall safety profile, followed by nivolumab (anti-PD1 antibody), pembrolizumab (anti-PD1 antibody) and ipilimumab (anti-CTLA4 antibody). The common AEs related to the clinical use of immune checkpoint inhibitors are summarized in **Table 3**.

**Table 3:** Summary of adverse events in immune checkpoint inhibition.

Agent	Adverse events
**Pembrolizumab**	**Arthralgia**, **Pneumonitis**, **Hepatotoxicity**, Autoimmune hepatitis, Fatigue, Pruritus, Rash, Diarrhea, Colitis, Nausea, Vomiting, Hypothyroidism, Hyperthyroidism
**Nivolumab**	**Endocrine toxicities**, Pneumonitis, Hepatitis, Diarrhea, Colitis, Fatigue, Pruritus, Nausea
**Atezolizumab**	**Fatigue**, **Hypothyroidism**, **Nausea**, **Vomiting**, Pruritus, Rash, Diarrhea, Pneumonitis, Arthralgia
**Ipilimumab**	**Skin**, **Gastrointestinal toxicities**, **Renal toxicities**, Autoimmune hepatitis, Fatigue, Diarrhea, Colitis, Nausea, Vomiting, Pneumonitis, Hypothyroidism, Hyperthyroidism, Arthralgia

The major adverse events are depicted in boldface.

## Concluding Remarks

Immune checkpoint molecules can prevent the excessive activation of T cells caused by inflammation in order to maintain their own tolerance. Tumor cells are able to activate these checkpoint molecules to suppress host’s immune response (61), thereby impairing immune surveillance and assault (62). However, early clinical trials have shown that tremelimumab monotherapy is inefficient and will lead to AEs such as skin rash, diarrhea, and endocrine abnormalities (63). Additionally, colitis may be caused by autoimmune-related mechanisms during treatment with CTLA4 blockade (64). Because of this and other shortcomings related with anti-CTLA4 inhibition, more studies are being focused on PD1/PDL1 inhibition. Hopefully, new CTLA4 inhibitors and/or combinations with better performance will be developed.

In addition, selection of those patients who would benefit from immunotherapy is of utmost importance and is a major challenge in considering immune checkpoint-based immunotherapy. Particularly, due consideration should be given to the different subtypes of TNBC. It is now well accepted that TNBC is a heterogeneous group of diseases comprising different subtypes with different histopathological and molecular makeups. TNBC can be grouped into six (21) or four subtypes (65, 66), depending on the classification system used. In general, the immunomodulatory (IM) subtype of TNBC possesses elevated infiltration of immune cells, and hence, is more likely to be responsive to immunotherapy (67). Additionally, basal-like TNBCs are deemed to have high frequency of BRCA1 and BRCA2 mutations and genetically unstable, which is another predictor of immunotherapy response (65, 68). Furthermore, since the immune checkpoint molecules (e.g., PDL1) is expressed in a portion of TNBC patients (69, 70), we need to be aware of the status of these molecules in TNBC patients in order to get optimized immunotherapeutic efficiency. Nevertheless, immune checkpoint-based therapies provide the opportunity of less toxicity and enhanced potency leading to durable and long-lasting responses for TNBC patients.

## Author Contributions

HY, YL, and YT: Investigation and writing—original draft. SF: Investigation, writing—review and editing. FT and XD: Writing—review and editing and supervision. All authors contributed to the article and approved the submitted version.

## Funding

This work was supported by the National Natural Science Foundation of China (81872167, 81472496, 81872226), the Natural Science Foundation of Hunan (2019JJ40193), Hunan Provincial Innovation Foundation for Postgraduate (CX2018B305), the Key Project of Department of Education of Hunan Province (14A089), and Changsha Municipal Natural Science Foundation (kq2014080).

## Conflict of Interest

The authors declare that the research was conducted in the absence of any commercial or financial relationships that could be construed as a potential conflict of interest.
